# Breast Cancer Stem Cell-Derived ANXA6-Containing Exosomes Sustain Paclitaxel Resistance and Cancer Aggressiveness in Breast Cancer

**DOI:** 10.3389/fcell.2021.718721

**Published:** 2021-10-05

**Authors:** Zihe Guo, Ayao Guo, Chuang Zhou

**Affiliations:** ^1^Department of Breast Surgery, The First Affiliated Hospital of China Medical University, Shenyang, China; ^2^Department of Oncology, The AnsSteel Group Hospital, Anshan, China

**Keywords:** breast cancer stem cell, annexin-A6, paclitaxel, cancer metastasis, autophagy

## Abstract

Continuous chemotherapy pressure-elicited annexin-A6 (ANXA6)-containing exosome (ANXA6-exo) secretion contributes to paclitaxel (PTX) resistance in breast cancer (BC), but the molecular mechanisms are not fully elucidated. The present study managed to investigate this issue and found that ANXA6-exo promoted PTX resistance and cancer progression in BC cells in a Yes-associated protein 1 (YAP1)-dependent manner. Specifically, the parental PTX-sensitive BC (PS-BC) cells were exposed to continuous low-dose PTX to generate PTX-resistant BC (PR-BC) cells, and we found that BC stem cells tended to be enriched in the descendent PR-BC cells in contrast with the PS-BC cells. In addition, PR-BC cell-derived exosomes were featured with highly expressed ANXA6, and ANXA6-exo delivered ANXA6 to promote cell migration, growth, autophagy, and stemness in PS-BC cells. Interestingly, ANXA6-exo increased PTX resistance in PS-BC cells *via* inducing autophagy, and the effects of ANXA6-exo on PTX resistance in PS-BC cells were abrogated by co-treating cells with the autophagy inhibitor 3-methyladenine. Moreover, the underlying mechanisms were uncovered, and we evidenced that ANXA6-exo up-regulated YAP1 to promote Hippo pathway dysregulation, and the promoting effects of ANXA6-exo on PTX resistance and cancer aggressiveness in BC cells were abrogated by silencing YAP1. Taken together, this study firstly elucidated the underlying mechanisms by which BCSC-derived ANXA6-exo facilitated BC progression and PTX resistance, which might help to develop novel treatment strategies for BC in clinic.

## Introduction

Paclitaxel (PTX) is a common chemical drug for breast cancer (BC) treatment (Yu et al., 2020; Schmid et al., 2020a, b); however, the therapeutic efficiency of this chemical drug is seriously limited as the results of PTX resistance have been a huge health burden for BC patients worldwide (Chi et al., 2019; Gupta et al., 2019). The underlying mechanisms by which BC cells generate resistant properties to PTX treatment are complicated, and researchers agree that cell stemness (Yan et al., 2018; Mori et al., 2019) and autophagy (Zhang et al., 2015; Datta et al., 2019) are two major factors that contribute to this process. As previously described (Yan et al., 2018; Mori et al., 2019), cancer stem cells (CSCs) are a group of cancer cells featured with self-renewal and are capable of differentiating into heterogeneous cancer cells, which are pivotal for sustaining drug resistance. Specifically, PTX treatment initially kills almost all the PTX-sensitive BC (PS-BC) cells, while a subgroup of BC cells with CSC properties survive, which further differentiate into cancer cells with PTX resistance (Yan et al., 2018; Mori et al., 2019). In addition, autophagy is an evolutionarily conserved process that protects cells from death under environmental stress, which is crucial for cellular homeostasis and contributes to drug resistance during cancer treatment (Zhang et al., 2015; Datta et al., 2019). For example, data from Xu et al. (2019), Liu et al. (2020) and Yang et al. (2020) evidence that blockage of autophagy efficiently promotes PTX sensitivity in BC.

Interactions among differential cancer cells contribute to the stability of tumor microenvironment, which make cancer cell population more resistant to the external stimulus, such as chemical drugs (Au Yeung et al., 2016; Zhang et al., 2020). It is believed that the interactions among cancer cells are achieved by two branches of methods, including direct cell-to-cell interactions and indirect exosome delivery method (Au Yeung et al., 2016; Zhang et al., 2020). According to recent publications, cancer cell-derived exosomes promote drug resistance in multiple cancer types, including non-small cell lung cancer (Zhang et al., 2018), gastric cancer (GC; Zhang et al., 2020), and BC (Han et al., 2020). For example, Kreger et al. (2016) report that BC cells treated with PTX promote cell survival and chemo-resistance, and Bigagli et al. (2019) find that BC-derived exosomes simultaneously modulate EMT/MET transition and chemo-sensitivity. Interestingly, BC cell-derived exosomes also affect cell stemness and autophagy, and Yang et al. (2021) evidence that chemotherapy-elicited exosomes promote BC stemness and chemo-resistance *via* activating the EZH2/STAT3 signaling pathway. Also, studies from other teams validate that exosomes from BC cells also regulate drug resistance in BC by promoting cell autophagy (Wang X. et al., 2019; Han et al., 2020; Wang B. et al., 2020).

According to existing information, cancer cell-derived exosomes contain various molecules, including microRNAs (Yang et al., 2017), circular RNAs (Wang X. et al., 2020), long non-coding RNAs (Dong et al., 2018), transcriptional factors, and proteins (Chen et al., 2018), which are pivotal for regulating cellular functions in the surrounding cells. Among all the cancer-associated genes, annexin-A6 (ANXA6) is a multifunctional intracellular scaffolding protein that is frequently detected in extracellular vesicles and is closely associated with cancer progression and drug resistance (Korolkova et al., 2020). Up until now, the role of ANXA6 in regulating cancer progression has not been well studied, and interestingly, Uchihara et al. (2020) evidence that cancer associated fibroblast-derived ANXA6-containing extracellular vesicles enhance drug resistance in BC, and Leca et al. (2016) support the idea that cancer-associated fibroblast-derived ANXA6-containing exosomes facilitate pancreatic cancer aggressiveness.

Thus, in the present study, we focused on investigating whether BC cells secreted ANXA6-containing exosomes to modulate PTX resistance and cancer progression in BC, and we evidenced that PTX-resistant BC (PR-BC) cells delivered ANXA6 to PS-BC cells through secreting ANXA6-containing exosomes, which further up-regulated its downstream oncogene Yes-associated protein 1 (YAP1), leading to cancer progression and PTX resistance in BC.

## Materials and Methods

### Clinical Specimen Collection

The BC patients (*N* = 74) in the First Affiliated Hospital of China Medical University from 2018 to 2020 were recruited and were divided into two groups with (*N* = 18) our without (*N* = 15) PTX treatment before surgical resection, and the BC tissues were collected and stored at -70°C conditions for further analysis. The written informed consent forms had been signed by all the participates, and all the clinical experiments were approved by the Ethics Committee of the First Affiliated Hospital of China Medical University (Grant No. 2018122601).

### Cells and Treatments

Human BC cell lines (MCF-7 and MDA-MB-231) were obtained from ATCC (United States), and the cells were authenticated by performing Short Tandem Repeat profiling. According to the experimental protocols from previous publications (Chi et al., 2019; Gupta et al., 2019), the BC cells were cultured in the RPMI 1640 medium (Gibco, United States) containing 10% fetal bovine serum (FBS, Gibco, United States) and were exposed to continuous low-dose PTX stimulation (from 0.1 to 5 μg/ml) for 6 months to generate MCF-7/PR and MDA-MB-231/PR cells, which were maintained in the medium with 5 μg/ml PTX. Finally, the BC/PR cells were subjected to high-dose PTX (50 μg/ml) and autophagy inhibitor 3-methyladenine (3-MA; 5 mM) treatment for further analysis.

### Delivery of Genes Manipulation Vectors

The gene manipulating vectors, including overexpression and downregulation vectors for ANXA6 and YAP1, were designed according to the previous publications, and were constructed and synthesized by Sangon Biotech (Shanghai, China). Briefly, the sequences for ANXA6 and YAP1 were cloned into the pcDNA3.1 overexpression vectors, and the small interfering RNAs were constructed. The above vectors were transfected into the BC cells through using the Lipofectamine Transfection reagent purchased from Invitrogen (United States) in keeping with the manufacturer’s protocol.

### Examination of Cell Proliferation and Viability

The BC cells were cultured in 96-well plates at a density of 800 cells per well, and the cells were subsequently subjected to high-dose PTX (50 μg/ml) for 0, 6, 12, 24, and 48 h, respectively. Then, 20 μl of MTT solution was added to each well of 96-well plates for 4 h at 37°C, which were further dissolved in 150 μl of DMSO and fully vortexed. A microplate reader (ThermoFisher Scientific, United States) was further used to examine the optical density (OD) values at 459 nm absorbance to represent cell proliferation abilities. At the same time, the cells were stained with trypan blue dye, and the dead blue cell numbers were counted under a light microscope (ThermoFisher Scientific, United States) to evaluate cell viability.

### Real-Time Quantitative Real-Time Analysis

Commercial TRIZol Reagent (Beyotime, Shanghai, China) was used for total RNA extraction, and the RNA quality was guaranteed by performing agarose electrophoresis. The complementary DNA was generated by using the HiScript II Q Select RT SuperMix Reagent (Vazyme, Nanjing, China), and quantitative real-time qPCR was conducted through the SYBR Green regents purchased from Vazyme (Nanjing, China) according to the producer’s protocols.

### Western Blot Analysis

The RIPA lysis buffer (Beyotime, Shanghai, China) was used to extract the total proteins, and its quality was measured by BCA kit (Beyotime, Shanghai, China). The proteins were separated by 10% SDS-PAGE, and the targeted proteins were transferred onto the PVDF membranes according to the proteins’ molecular weight. Membranes were then blocked with 5% non-fat milk and were incubated with the primary antibodies against LC3B (1:2000, Santa Cruz, United States), p62 (1:2000, Santa Cruz, United States), GAPDH (1:2000, Takara, Japan), ANXA6 (1:2000, Santa Cruz, United States), TSG101 (1:1500, Takara, Japan), Alix (1:1500, Takara, Japan), and HSP70 (1:1000, Santa Cruz, United States) at 4°C overnight. Then, the PVDF membranes were cultivated with the secondary HRP antibodies (ThermoFisher Scientific, United States) for 2 h at 37°C, and the ECL system was used to visualize the protein bands, which were quantified and analyzed by using the Image J software.

### Immunofluorescent Staining Assay

The MCF-7 and MDA-MB-231 cells with or without PTX treatments were fixed by using 4% paraformaldehyde, and the cells were permeabilized through exposing the cells to 0.5% Triton X-100 for 40 min at room temperature. Then, the cells were stained with anti-LC3B primary antibody (1:1,000, Santa Cruz, United States) and Alexa Fluro594 secondary antibody (1:2,000, Life Technologies, United States). The nucleus of the cells was stained by 4’,6-diamidino-2-phenylindole (DAPI), and a confocal imaging system (Olympus Microsystems, Japan) was used to examine the expression levels of localization of LC3B in the BC cells.

### Spheroid Formation Assay

The PS-BC and PR-BC cells were seeded onto 96-well plates at a density of 1,000 cells per well, and the cells were cultured in the RPMI 1640 medium (Gibco, United States) supplemented with 20 ng/ml epithelial growth factor, 10 ng/ml fibroblast growth factor-2, and 100 μg/ml penicillin G in an incubator with standard culture conditions with 5% CO_2_ humidified air at 37°C. At 7 days post-culture, the sphere diameters above 40 μm were counted under a light microscope (ThermoFisher Scientific, United States).

### Measurement of Cell Death Through Flow Cytometry

Breast cancer cell death ratio was examined by using the Dead Cell Detection kit purchased from Cell Signaling Technology (MA, United States) according to the producer’s protocol. Briefly, the BC cells were fixed by using the 4% paraformaldehyde for 15 min at room temperature, and the cells were subsequently stained with Annexin V-FITC (10 μl) and propidium iodide (5 μl) on ice without light exposure. Finally, cell death was measured by using the flow cytometer and analyzed by the FlowJo 7.6.1 software (FlowJo LLC, United States).

### Transwell Assay for Cell Migration

Breast cancer cells were seeded onto the upper chamber of the 6-well BD Migration Chambers (BD Bioscience, United States) at a density of 1 × 10^5^ cells per well with serum-free RPMI 1640 medium (Gibco, United States), and the lower chamber was added with the medium containing 10% fetal bovine serum (FBS, Gibco, United States). At 48 h post-culture, the cells in the surface were removed and the migration cells were stained with crystal violet for 15 min at room temperature, which were further counted under a light microscope (ThermoFisher Scientific, United States).

### Xenograft Tumor-Bearing Mice Models

Male BALB/c nude mice (*N* = 6) were purchased from the experimental animal center of China Medical University and were maintained in the conditions with specific-pathogen free level. The MCF-7 cells were pre-incubated with ANXA6-negative and -positive exosomes for 12 h, and the cells were subcutaneously injected into the dorsal flank of the mice at the concentration of 5 × 10^6^ cells per mice. The mice were anesthetized and sacrificed at day 20 when the tumor volume in the ANXA6-positive group reached about 100 mm^3^, and the tumors were obtained, weighed, and stored at -70°C conditions for further analysis. All the animal experiments were approved by the Ethics Committee of the First Affiliated Hospital of China Medical University (Grant No. 2017A023103).

### Immunohistochemistry for Ki67 Expressions and Localization

The mouse tumor tissues were sliced into sections with 5-μm thickness, fixed with 4% paraformaldehyde, and embedded with paraffin. The sections were then deparaffinized, subjected to a graded series of ethanol for rehydration, and heated for antigen retrieval. After that, the tissues were washed by PBST buffer and blocked with normal goat serum, and the sections were incubated with primary Ki67 antibody (Abcam, United Kingdom) at 4°C overnight and the secondary antibody for 2 h at 37°C. Then, the sections were stained with diaminobenzidine and counterstained with hematoxylin for visualization, and a digital microscope (Nikon, Japan) was used to observe the yellow Ki67-positive cells in mouse tumor tissues.

### Isolation, Purification, and Observation of Breast Cancer Cell-Derived Exosomes

The BC cells were cultured in the incubator for 48 h, and the supernatants were harvested, which were subsequently subjected to centrifugation procedures, including 500 *g* for 5 min, 2,000 *g* for 15 min, and 10,000 *g* for 20 min at 4°C, and the supernatants without cells, debris, and large vesicles were obtained. Then, the supernatants were centrifuged at 140,000 *g* for 70 min, and the pellet was resolved in PBS for further analysis. Then, the size and concentration of the exosomes were determined by Nanoparticle Tracking Analysis, which were observed and photographed by performing transmission electron microscopy. Briefly, the exosomes were applied to carbon-coated 400 mesh grids, washed by PBS, and stained with 2% uranyl acetate for 30 s, and images were captured by using the electron microscope device (Tecnai Spirit, FEI Company, ThermoFisher Scientific, United States).

### Statistical Analysis

Analysis of the data was performed by using the SPSS 18.0 software and GraphPad Prism 8.0 software. The means from two groups were compared by Student’s *t*-test, and means from multiple groups were analyzed by one-way ANOVA analysis. Also, Pearson correlation analysis was performed to analyze the correlations among the cancer-associated genes in clinical tissues. *p* < 0.05 was regarded as statistical significance, and were indicated by “*.”

## Results

### The Association of Cell Autophagy and Stemness With Paclitaxel Resistance in Breast Cancer

**FIGURE 1:**
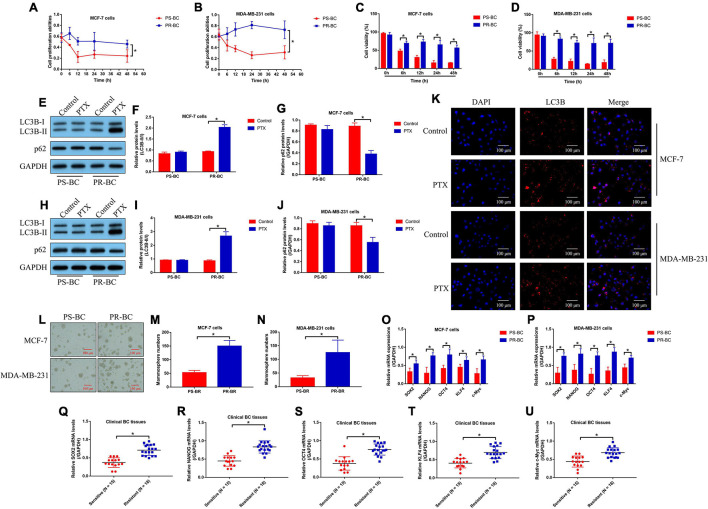
Cell autophagy and stemness were closely relevant to PTX resistance in BC. **(A,B)** Cell proliferation and **(C,D)** viability were, respectively, determined by MTT assay and trypan blue staining assay. **(E–J)** The autophagy-associated proteins were quantified by performing Western blot analysis. **(K)** The expression levels and localization of LC3B protein in BC cells were determined by immunofluorescent staining assay. **(L–N)** Spheroid formation assay was used to examine spheroid formation abilities in BC cells. The mRNA levels of SOX2, NANOG, OCT4, KLF4, and c-Myc in **(O,P)** BC cells and **(Q–U)** clinical tissues were analyzed by performing real-time qPCR. Single experiment had three repetitions, and *p < 0.05 was regarded as statistical significance.

As previously described (Chi et al., 2019; Gupta et al., 2019), the PS-BC cells (MCF-7 and MDA-MB-231) were exposed to continuous low-dose PTX (from 0.1 to 5 μg/ml) treatment to generate PR-BC cells (MCF-7/PR and MDA-MB-231/PR), which were subsequently subjected to high-dose PTX (50 μg/ml) stimulation. The MTT assay (Figures 1A,B) and trypan blue staining assay (Figures 1C,D) results showed that PR-BC cells were much more resistant to high-dose PTX in contrast with their parental PS-BC cells, suggesting that the PR-BC cells with PTX-resistant properties had been successfully established. To our knowledge, cell autophagy and stemness are two major factors that contribute to chemo-resistance in multiple cancers (Zhang et al., 2015; Yan et al., 2018; Datta et al., 2019; Mori et al., 2019), which encouraged us to investigate whether PTX induced chemo-resistance in BC cells in a similar manner. To explore this issue, the PS-BC cells and PR-BC cells were initially subjected to high-dose PTX (50 μg/ml) exposure for 24 h, and we surprisingly found that PTX increased the LC3B-II/I ratio and down-regulated p62 in PR-BC cells, instead of the PS-BC cells (Figures 1E–J). Consistently, the following immunofluorescence staining assay results hinted that LC3B was up-regulated in PTX-treated PR-BC cells (Figure 1K). Next, CSC properties in PS-BC and PR-BC cells were compared. Specifically, by performing the spheroid formation assay, we noticed that PR-BC cells were prone to form mammospheres compared with the PS-BC cells (Figures 1L–N). In addition, the real-time qPCR analysis results supported that the mRNA levels of stemness-related genes, including SOX2, NANOG, OCT4, KLF4, and c-Myc, were all elevated in the PR-BC cells, compared to the corresponding PS-BC cells (Figures 1O,P). Furthermore, we collected the PTX-resistant (*N* = 18) and PTX-sensitive (*N* = 15) BC tissues in our hospital, and the data in Figures 1Q–U indicated that SOX2, NANOG, OCT4, KLF4, and c-Myc mRNA levels were up-regulated in PR-BC tissues. The above data suggested that cell autophagy and stemness were closely relevant to PTX resistance in BC.

### Paclitaxel-Resistant Breast Cancer-Derived Annexin-A6-Containing Exosomes Promoted Paclitaxel Resistance in Breast Cancer Cells

**FIGURE 2:**
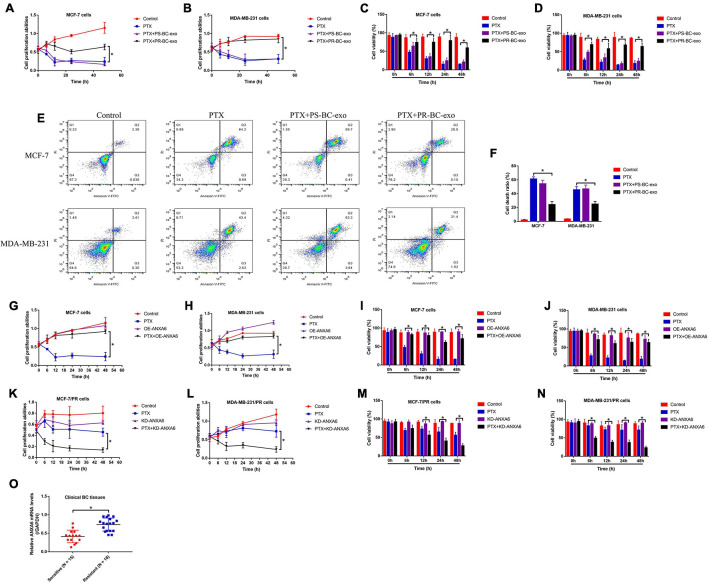
BCSC-derived ANXA6-containing exosomes increased PTX resistance in PS-BC cells. The PS-BC cells were co-cultured with ANXA6 exosomes, and **(A,B)** MTT assay and **(C,D)** trypan blue staining assay were used to measure cell proliferation and viability. **(E,F)** BC cells were stained with PI and Annexin V-FITC, and FCM was subsequently used to examine cell death ratio. Cell proliferation and viability in **(G–J)** PS-BC cells and **(K–N)** PR-BC cells were, respectively, examined. **(O)** The mRNA levels of ANXA6 in BC tissues were measured by real-time qPCR. A single experiment had three repetitions, and *p < 0.05 was regarded as statistical significance.

Previous publications report that BC stem cells (BCSCs) were capable of interacting with the surrounding BC cells by secreting exosomes, which also influences chemo-resistance of chemical drugs during BC clinical treatments (Au Yeung et al., 2016; Zhang et al., 2020). Thus, we isolated the PS-BC and PR-BC cell-derived exosomes (PS-BC-exo and PR-BC-exo), which were photographed by EM (Supplementary Figure 1A), and the following Western blot analysis results validated that the exosome-associated biomarkers (Alix, HSP70 and TSG101) were enriched in those exosomes (Supplementary Figure 1B). Then, the PS-BC cells were, respectively, pre-incubated with the PS-BC-exo and PR-BC-exo for 24 h, and the cells were subsequently stimulated with high-dose PTX (50 μg/ml). Interestingly, the MTT assay (Figures 2A,B), trypan blue staining assay (Figures 2C,D), and Flow cytometry (FCM) assay (Figures 2E,F) results supported that PR-BC-exo but not PS-BC-exo promoted cell proliferation and viability, and suppressed cell death to increase PTX resistance in PS-BC cells. Next, by searching the existing information from the online PubMed database, we noticed that one of the exosomal protein ANXA6 is closely associated with cancer progression (Kapustin and Shanahan, 2016; Keklikoglou et al., 2019) and drug resistance (Uchihara et al., 2020), and this protein also plays an important role in regulating the biological functions of BC cells during its pathogenesis (Sakwe et al., 2011; Widatalla et al., 2019; Korolkova et al., 2020). By performing Western blot analysis, we surprisingly found that ANXA6 tended to be enriched in PR-BC-exo compared to the PS-BC-exo, and the relative protein levels were normalized by using the exosome marker TSG101 (Supplementary Figures 2A,B). Also, the expression levels of ANXA6 in PR-BC cells were much higher than that of PS-BC cells (Supplementary Figures 2C–E), and PR-BC-exo delivered ANXA6 protein for its upregulation in PS-BC cells (Supplementary Figures 2F–H), which encouraged us to select exosomal ANXA6 for further investigation. Then, ANXA6 was silenced in PR-BC cells (Supplementary Figure 2I) and was overexpressed in PS-BC cells (Supplementary Figure 2J); the BC cells were subsequently treated with high-dose PTX. Our data suggested that ANXA6 overexpression made PS-BC cells more resistant to high-dose PTX treatment (Figures 2G–J), and conversely, knockdown of ANXA6 increased PTX sensitivity in PR-BC cells (Figures 2K–N). Moreover, ANXA6 was up-regulated in the BC tissues collected from BC patients with PTX resistance (Figure 2O).

### Influences of Paclitaxel-Resistant Breast Cancer Cell-Derived Annexin-A6-Exo on Cell Migration, Growth, and Stemness in Paclitaxel-Sensitive Breast Cancer Cells

**FIGURE 3:**
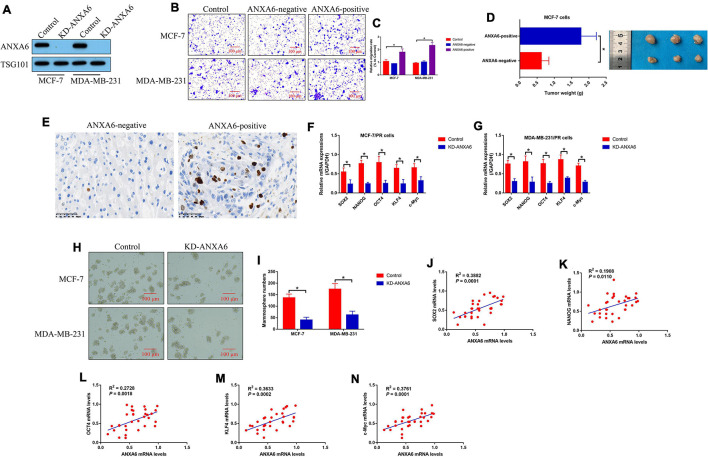
BCSC-secreted exosomes promoted tumor migration, growth, and stemness via delivering ANXA6. **(A)** ANXA6 was silenced in PR-BC-derived exosomes, which were verified by Western blot analysis. The BCSC-derived exosomes with or without ANXA6 were co-cultured with the PS-BC cells. **(B,C)** Cell migration abilities were examined by Transwell assay. **(D)** *In vivo* BC cell growth was monitored, and **(E)** Ki67 protein levels in mouse tumor tissues were measured by immunohistochemistry (IHC) assay. **(F,G)** Cell stemness-associated proteins were determined by real-time qPCR, and **(H,I)** spheroid formation abilities in BC cells were determined by spheroid formation assay. **(J–N)** The correlations between ANXA6 mRNA and stemness-associated biomarkers in clinical BC tissues were analyzed by performing Pearson correlation analysis. A single experiment had three repetitions, and *p < 0.05 was regarded as statistical significance.

Since previous data hint that ANXA6 acts as an oncogene to aggravate malignancy in BC, we investigated whether PR-BC-exo affected the malignant phenotypes, including cell mobility, growth, and stemness in BC cells. The silencing vectors for ANXA6 were transfected into the PR-BC cells, and the Western blot analysis results evidenced that we successfully ablated ANXA6 in PR-BC-exo (Figure 3A). Then, the ANXA6-negative and positive PR-BC-exo were pre-incubated with PS-BC cells for 12 h, and the Transwell assay results showed that ANXA6-positive PR-BC-exo significantly promoted cell migration in PS-BC cells (Figures 3B,C). Next, the above PS-BC cells were injected into the mice dorsal flanks to establish tumor-bearing mice models *in vivo*, and the results in Figure 3D showed that PR-BC-exo promoted tumorigenesis of MCF-7 cells by delivering ANXA6, which were supported by the Immunohistochemistry (IHC) assay results that ANXA6-positive PR-BC-exo also increased Ki67 protein levels in mouse tumor tissues (Figure 3E). Moreover, we evidenced that ANXA6 controlled CSC properties in PR-BC cells. Specifically, knockdown of ANXA6 suppressed mRNA levels of SOX2, NANOG, OCT4, KLF4, and c-Myc in PR-BC cells (Figures 3F,G), and ANXA6 ablation also restrained PR-BC cells’ spheroid formation abilities (Figures 3H,I). Finally, we analyzed the correlations of ANXA6 with cell stemness-associated biomarkers in clinical specimens, and expectedly found that ANXA6 positively correlated with SOX2 (Figure 3J), NANOG (Figure 3K), OCT4 (Figure 3L), KLF4 (Figure 3M), and c-Myc (Figure 3N) in BC tissues (*N* = 33).

### Annexin-A6 Upregulation Promoted Paclitaxel Resistance in Paclitaxel-Sensitive Breast Cancer Cells *via* Inducing Protective Autophagy

**FIGURE 4:**
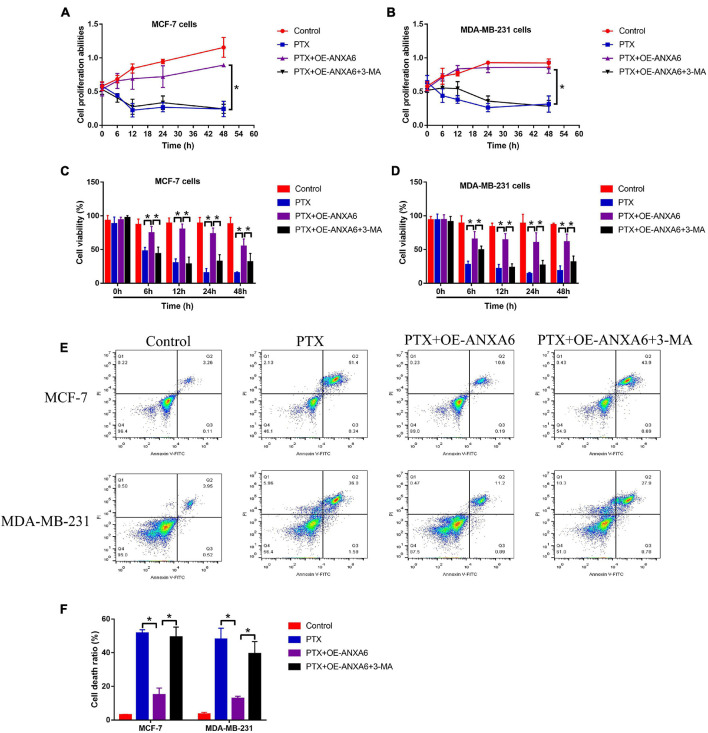
Upregulation of ANXA6 increased PTX resistance in PS-BC cells by triggering protective autophagy. **(A,B)** MTT assay and **(C,D)** trypan blue staining assay were performed to evaluate cell proliferation and viability in PS-BC cells. **(E,F)** Cell death ratio was measured by using the FCM assay. A single experiment had three repetitions, and *p < 0.05 was regarded as statistical significance.

Given that ANXA6 induces protective autophagy (Chen et al., 2020; Sun X. et al., 2020), and induction of cell autophagy contributes to PTX resistance in BC cells (Zhang et al., 2015; Datta et al., 2019), we next explored whether ANXA6 overexpression promoted PTX resistance in PS-BC cells *via* activating autophagy flux. To achieve this, the PS-BC cells were subjected to ANXA6 overexpression vector (OE-ANXA6) transfection (Supplementary Figure 2J) and autophagy inhibitor (3-MA), which were subsequently stimulated with high-dose PTX (50 μg/ml). The cells were divided into four groups: Control, PTX-alone group, PTX + OE-ANXA6 group, and PTX + OE-ANXA6 + 3-MA group. Results in Supplementary Figures 5A–F showed that ANXA6-induced cell autophagy in PS-BC cells was successfully blocked by 3-MA. Additionally, the MTT assay results showed that 3-MA alone did not influence cell proliferation in PS-BC cells (Figures 4A,B). Interestingly, 3-MA significantly abrogated the promoting effects of ANXA6 overexpression on cell proliferation in PTX-treated PS-BC cells (Figures 4A,B). Similarly, the trypan blue staining assay (Figures 4C,D) and FCM assay (Figures 4E,F) data supported the idea that overexpression of ANXA6 rescued cell viability and restrained cell death in PTX-treated PS-BC cells, which were all reversed by blocking protective autophagy *via* 3-MA. The above data suggested that ANXA6 increased PTX resistance in PS-BC cells in an autophagy-dependent manner.

### Upregulation of Annexin-A6 Promoted Cell Migration, Stemness, and Autophagy in Paclitaxel-Sensitive Breast Cancer Cells Through Yes-Associated Protein 1

**FIGURE 5:**
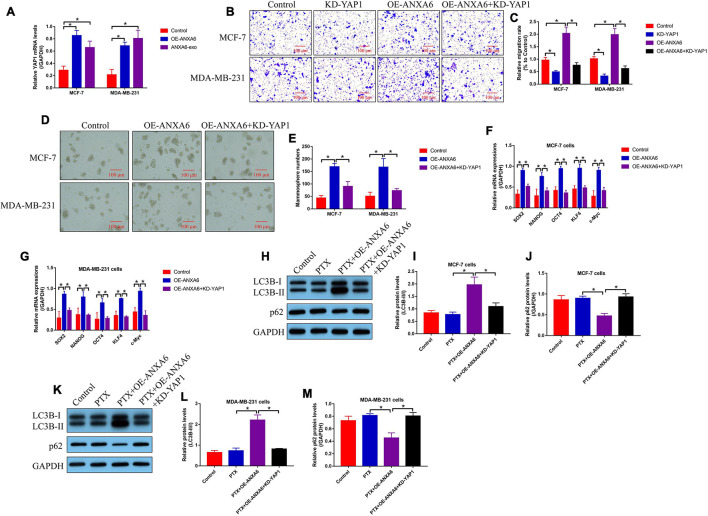
ANXA6 overexpression promoted cell migration, stemness, and autophagy in PS-BC cells through upregulating YAP1. **(A)** Real-time qPCR analysis validated that ANXA6 positively regulated YAP1 mRNA levels in PS-BC cells. **(B,C)** Transwell assay was used to examine cell migration abilities. Cancer stem cell properties in BC cells were evaluated by **(D,E)** spheroid formation assay and **(F,G)** real-time qPCR analysis. **(H–M)** The regulating effects of ANXA6 and YAP1 on cell autophagy were investigated by performing Western blot analysis. A single experiment had three repetitions, and *p < 0.05 was regarded as statistical significance.

We next investigated the underlying mechanisms by which ANXA6 regulated cancer progression, cell stemness, and autophagy in BC cells, and according to the existing information that ANXA6 directly induces YAP1 activation (Uchihara et al., 2020), which is reported to be closely associated with BC development (Gan et al., 2019; Lee et al., 2019), we speculated that ANXA6 might exert its tumor-promoting role through modulating the YAP1/Hippo pathway. As shown in Figure 5A, the mRNA levels of YAP1 were increased by both ANXA6 upregulation and ANXA6-containing exosomes in PS-BC cells, suggesting that ANXA6 modulated DNA-to-mRNA transcription of YAP1 for its upregulation in BC cells. In addition, YAP1 was down-regulated in PS-BC cells (Supplementary Figures 3A,B), and Transwell assay results in Figures 5B,C showed that the promoting effects of ANXA6 overexpression on cell migration were abolished by silencing YAP1. Also, we evidenced that CSC properties in PS-BC cells were modulated by the ANXA6/YAP1 pathway in a similar manner. Specifically, overexpression of ANXA6 promoted spheroid formation in PS-BC cells, which were reversed by silencing YAP1 (Figures 5D,E). Also, ANXA6 positively regulated cell stemness-associated biomarkers (SOX2, NANOG, OCT4, KLF4, and c-Myc) in PS-BC cells, which were suppressed by knocking down YAP1 (Figures 5F,G). Moreover, YAP1-induced autophagy plays a pivotal role to induce drug resistance in multiple cancers (Zhu et al., 2018; Wang C. Z. et al., 2019), and we expectedly evidenced that ANXA6 overexpression-induced upregulation of LC3B-II/I ratio and p62 downregulation in PTX-treated PS-BC cells were all reversed by silencing YAP1 (Figures 5H–M).

### Silencing of Annexin-A6 Increased Paclitaxel Sensitivity in the Paclitaxel-Resistant Breast Cancer Cells *via* Inactivating Yes-Associated Protein 1

**FIGURE 6:**
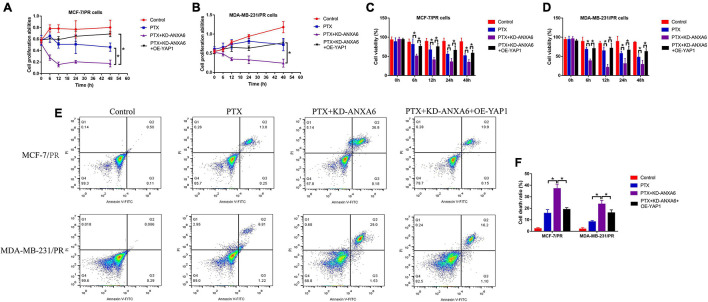
ANXA6 knockdown promoted PTX sensitivity in PR-BC cells via modulating YAP1. **(A,B)** MTT assay was used to detect cell proliferation. **(C,D)** Trypan blue staining assay was conducted to measure cell viability. **(E,F)** Cell death ratio was determined by performing FCM assay. A single experiment had three repetitions, and *p < 0.05 was regarded as statistical significance.

Given that we had proved that ANXA6 up-regulated YAP1 to regulate cell stemness and autophagy, which were pivotal for the generation of PTX resistance in BC, and ANXA6 directly regulated PTX resistance in BC, we hypothesized that the ANXA6/YAP1 pathway was also involved in regulating PTX sensitivity in BC. To validate our speculation, PR-BC cells were subjected to ANXA6 downregulation, YAP1 upregulation (Supplementary Figures 4A,B), and high-dose PTX treatments. The cells were divided into groups as follows: Control, PTX-alone group, PTX + KD-ANXA6 group, and PTX + KD-ANXA6 + OE-YAP1 group. As shown in Figures 6A–D, the MTT assay and trypan blue staining assay results showed that the inhibiting effects of ANXA6 ablation on cell proliferation and viability in PTX-treated PR-BC cells were reversed by overexpressing YAP1. In addition, the FCM assay results supported that YAP1 overexpression also suppressed PTX-induced cell death in PR-BC cells with ANXA6 overexpression (Figures 6E,F), implying that knockdown of ANXA6 increased susceptibility of PR-BC cells to high-dose PTX treatment *via* downregulating YAP1.

## Discussion

Drug resistance is a common phenomenon in clinic during cancer treatments, and PTX resistance has become a huge obstacle, which makes this chemical drug ineffective for BC treatment (Chi et al., 2019; Gupta et al., 2019); however, the unknown underlying mechanisms seriously limit our understanding in this field, which hampers the development of novel treatment strategies for BC. According to existing information (Zhang et al., 2015; Yan et al., 2018; Datta et al., 2019; Mori et al., 2019), cell autophagy and stemness are two major factors that contribute to drug resistance in various cancers, including BC, which are supported by the data in this study. Specifically, we evidenced that BCSCs were significantly enriched in the PR-BC cell population, instead of their corresponding parental PS-BC cells. Also, further experiments validated that high-dose PTX specifically triggered cell autophagy in PR-BC cells but not in PS-BC cells. The above results suggested that cell autophagy and stemness helped BC cells to generate PTX-resistant properties. Specifically, on the one hand, PR-BC cells are featured with CSC properties, which differentiate into PTX-resistant subpopulations under high-dose PTX pressure. On the other, high-dose PTX activates autophagy flux in PR-BC cells, which makes PR-BC cells much more resistant to PTX treatment compared to the PS-BC cells. The above data were supported by the previous publications (Zhang et al., 2015; Yan et al., 2018; Datta et al., 2019; Mori et al., 2019).

Interactions among differential types of cancer cells play important role in regulating cancer progression and drug resistance (Au Yeung et al., 2016; Zhang et al., 2020), and according to recent publications (Bigagli et al., 2019), cancer cells exchange “information” through secreting intracellular vesicles, such as exosomes. Based on existing information, this study evidenced that PR-BC cell-derived exosomes were capable of increasing PTX resistance in PS-BC cells, which were partially supported by previous literatures (Au Yeung et al., 2016; Bigagli et al., 2019; Zhang et al., 2020). To our knowledge, cancer cell-derived exosomes regulate cellular functions by delivering cancer-associated contents, including non-coding RNAs and cancer-associated proteins (Au Yeung et al., 2016; Zhang et al., 2020). Among all the proteins, ANXA6 is considered to be closely associated with cancer progression, which is frequently observed in extracellular vesicles (Korolkova et al., 2020). In this study, we evidenced that PR-BC cells secreted exosomes to promote PS-BC cell autophagy, stemness, migration, and growth by delivering ANXA6. Moreover, upregulation of ANXA6 increased PTX resistance in PS-BC cells through activating autophagy flux, which were supported by previous literatures that show that blockage of autophagy is effective to increase drug sensitivity in BC (Wen et al., 2020; Sun et al., 2021; Yu et al., 2021), implying that PR-BC-derived ANXA6-containing exosomes increased PTX resistance by inducing autophagy and CSC properties.

Yes-associated protein 1 is identified as an oncogene in multiple cancers (Kandasamy et al., 2020; Tsuji et al., 2020; Zhou et al., 2020), which is also closely associated with PTX resistance (Pan et al., 2016; He et al., 2019). In addition, YAP1 affects multiple cellular functions, including cell autophagy (Yao et al., 2020; Yuan et al., 2020) and stemness (Lu et al., 2018; Sun H. L. et al., 2020). For example, Yao et al. find that YAP1 triggers autophagy to promote cisplatin resistance in GC (Yao et al., 2020), and Lu et al. report that YAP1 interacts with FGFR1 to maintain cancer stem-like cell properties in lung cancer (Lu et al., 2018). Of note, ANXA6 influences the YAP1 pathway to regulate drug resistance (Uchihara et al., 2020), and this study verified that ANXA6 positively regulated YAP1 at mRNA levels, indicating that ANXA6 promoted YAP1 expressions through modulating its transcription process. Further experiments evidenced that YAP1 knockdown suppressed cell migration, stemness, and autophagy in ANXA6-overexpressed PS-BC cells, and consistently, upregulation of YAP1 abrogated the inhibiting effects of ANXA6 knockdown on PTX resistance in PR-BC cells, suggesting that ANXA6 regulated the cellular functions to promote PTX resistance in BC cells through modulating YAP1.

Taken together, this study investigated the potential underlying mechanisms by which BCSCs regulated PTX resistance in BC and verified that BCSC-derived exosomes delivered ANXA6 to the surrounding PS-BC cells, which further activated the YAP1-associated signaling pathways and promoted cell autophagy and stemness, leading to the generation of PTX resistance in PS-BC cells. Those lines of evidence firstly explored the interactions among different subgroups of BC cells that contributed to drug resistance in BC, which provided novel insights into our understanding of BC progression and drug resistance.

## Data Availability Statement

The original contributions presented in the study are included in the article/Supplementary Material, further inquiries can be directed to the corresponding author/s.

## Ethics Statement

The studies involving human participants were reviewed and approved by Ethics Committee of The First Affiliated Hospital of China Medical University. The patients/participants provided their written informed consent to participate in this study. The animal study was reviewed and approved by Ethics Committee of The First Affiliated Hospital of China Medical University.

## Author Contributions

ZG was responsible for conception, investigations, and manuscript drafting. CZ collected and analyzed the data, and provided technical support. AG designed this research, provided guidance, and proofread the final version of the manuscript. All authors contributed to the article and approved the submitted version.

## Conflict of Interest

The authors declare that the research was conducted in the absence of any commercial or financial relationships that could be construed as a potential conflict of interest.

## Publisher’s Note

All claims expressed in this article are solely those of the authors and do not necessarily represent those of their affiliated organizations, or those of the publisher, the editors and the reviewers. Any product that may be evaluated in this article, or claim that may be made by its manufacturer, is not guaranteed or endorsed by the publisher.
