# Supplementary material for: Breast Cancer Stem Cell-Derived ANXA6-Containing Exosomes Sustain Paclitaxel Resistance and Cancer Aggressiveness in Breast Cancer
Source: Front Cell Dev Biol. 2021 Oct 5;9:718721. doi: 10.3389/fcell.2021.718721 (PMC8523856; doi:10.3389/fcell.2021.718721)
Supplement: Supplementary file 1 [file Data_Sheet_1.docx]

**Supplementary Figures and Figure captions**

**
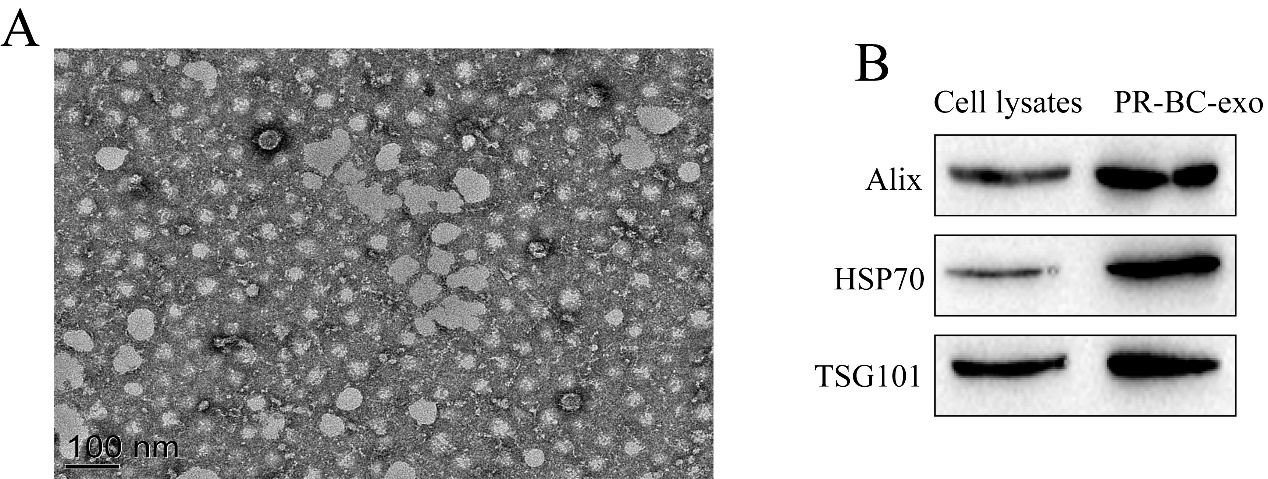
**

**Figure S1.** Isolation and purification of BCSCs-derived exosomes, which were observed by (A) EM and validated by performing (B) Western Blot analysis to examine the exosomes-associated biomarkers Alix, HSP70 and TSG101.


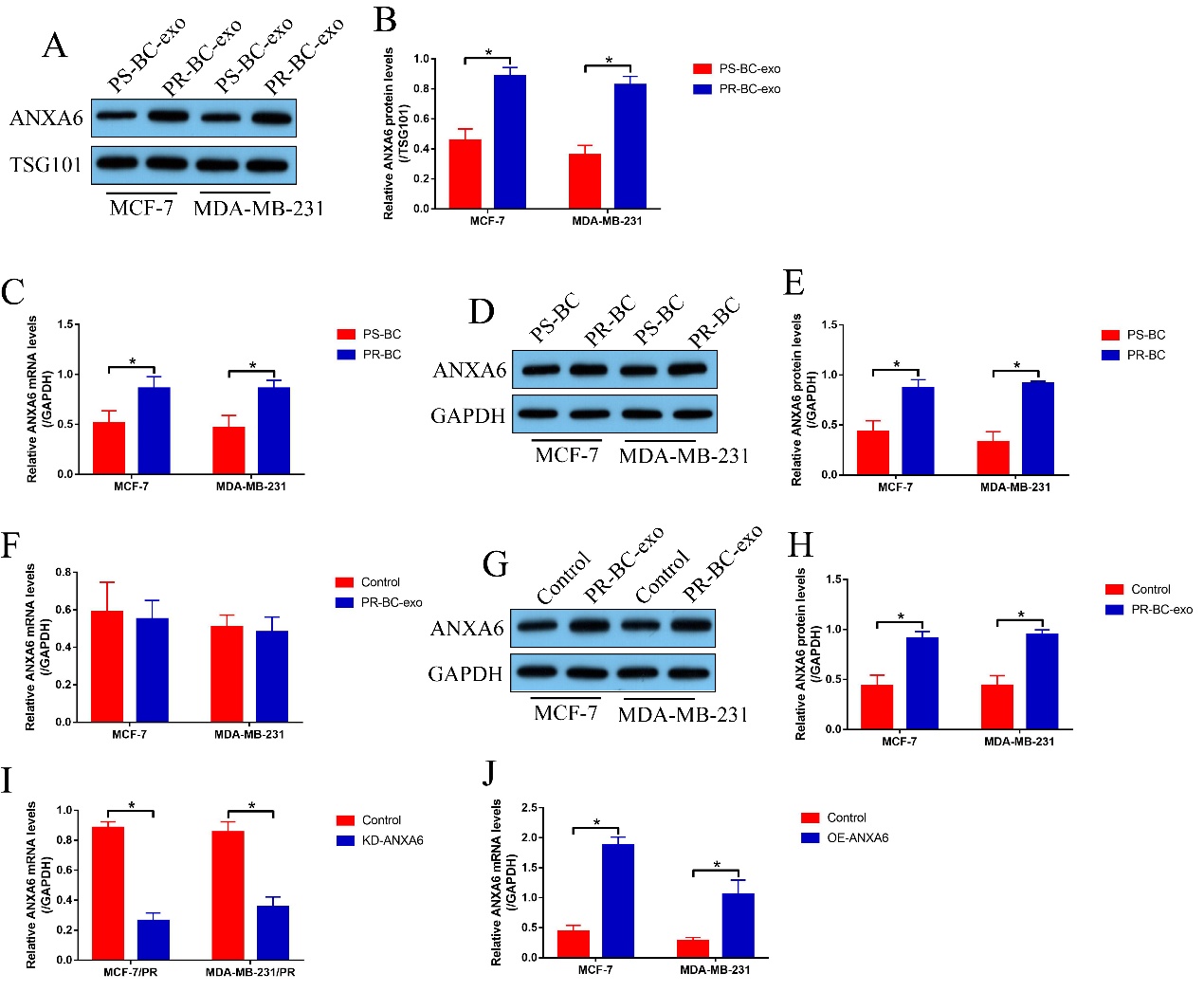


**Figure S2.** The mRNA and protein level of ANXA6 were respectively measured by using the (C, F, I, J) Real-Time qPCR and (A, B, D, E, G, H) Western Blot analysis. Single experiment had three repetitions, and **P* < 0.05 was regarded as statistical significance.


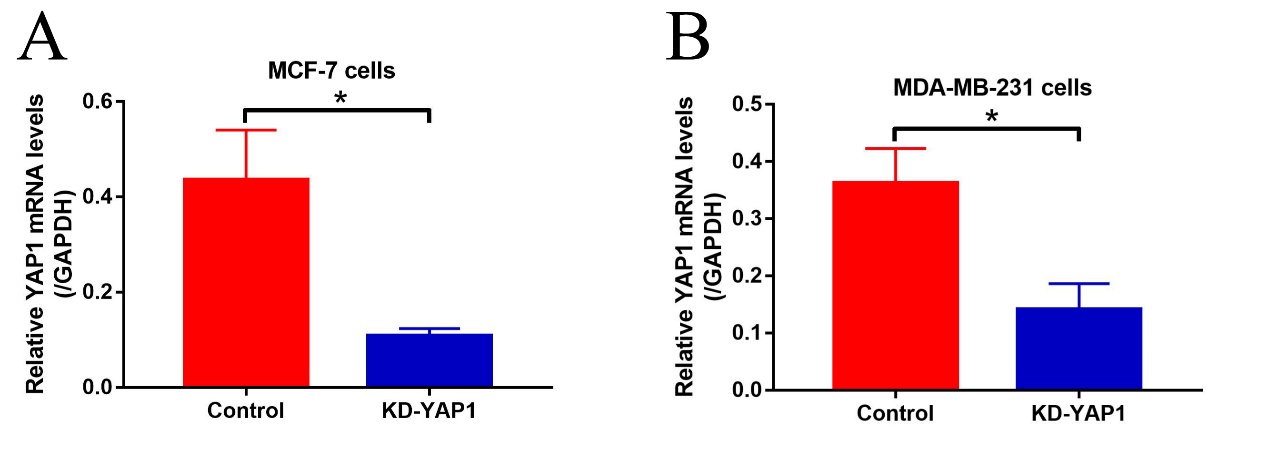


**Figure S3.** Real-Time qPCR was performed to detect YAP1 mRNA levels in MCF-7 and MDA-MB-231 cells. Single experiment had three repetitions, and **P* < 0.05 was regarded as statistical significance.


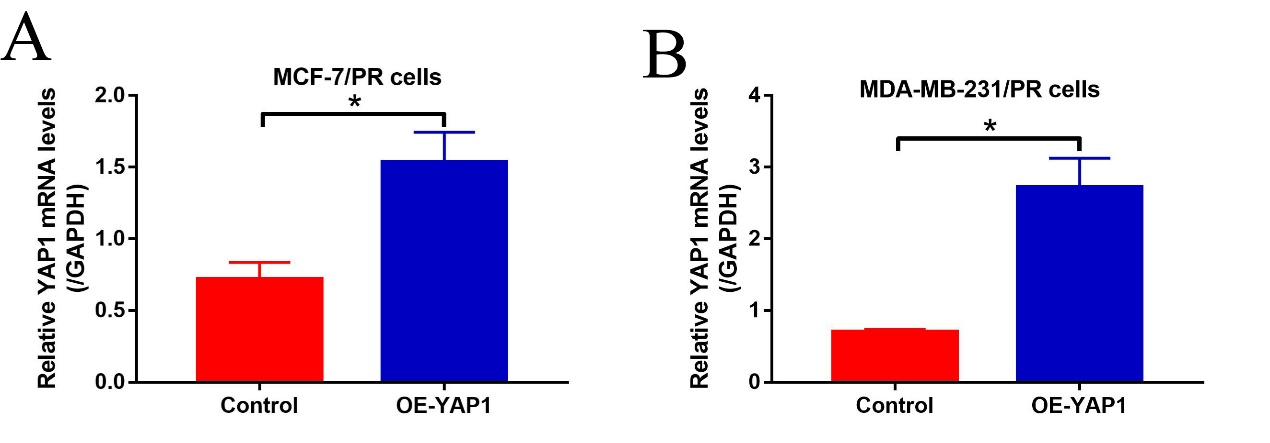


**Figure S4.** The mRNA levels of YAP1 in MCF-7/PR and MDA-MB-231/PR cells were examined by performing Real-Time qPCR analysis. Single experiment had three repetitions, and **P* < 0.05 was regarded as statistical significance.


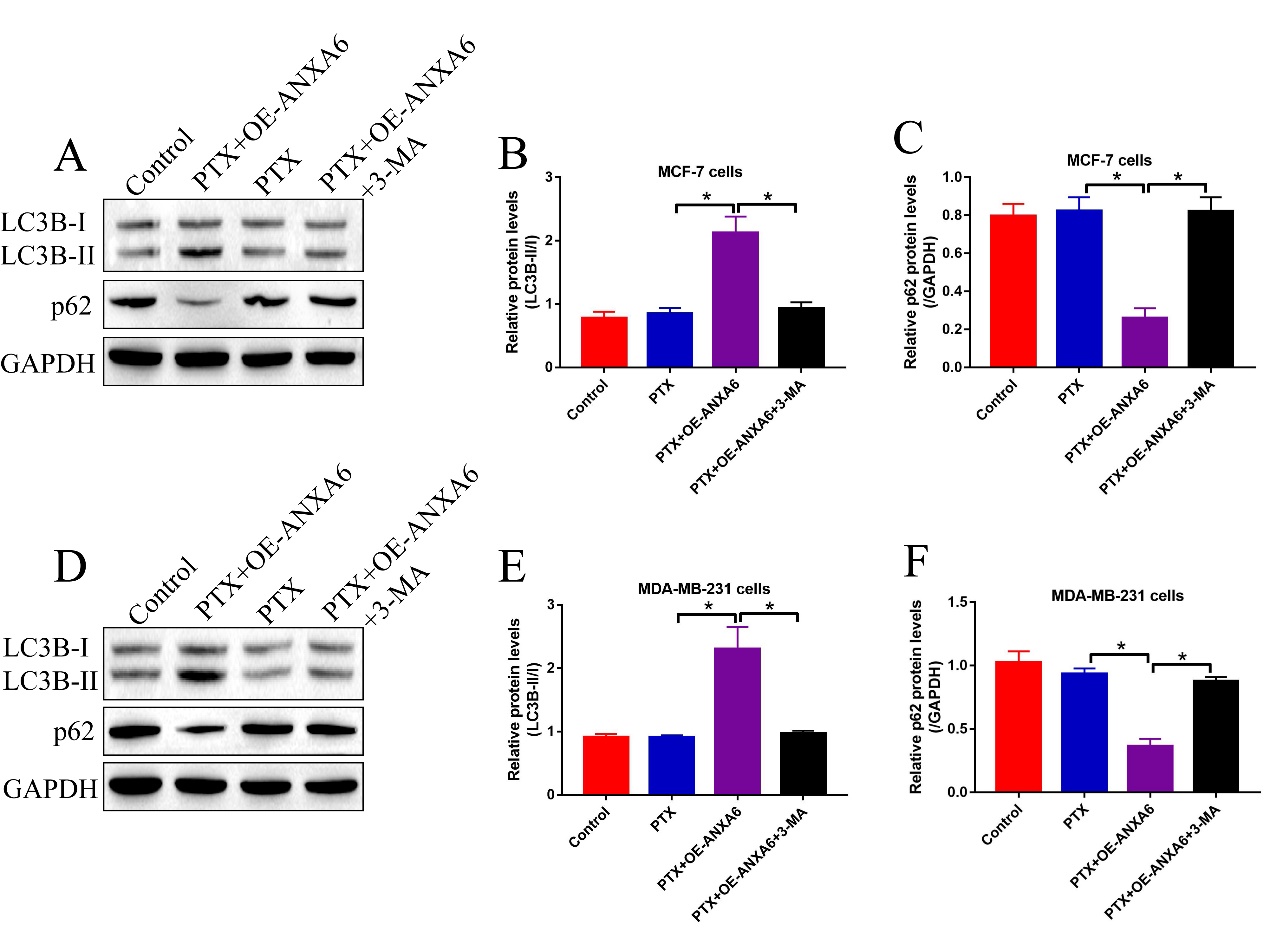


**Figure S5.** Western Blot analysis was performed to examine the expression status of autophagy-associated biomarkers (LC3B II/I and p62) in the PS-BC cells. Single experiment had three repetitions, and **P* < 0.05 was regarded as statistical significance.
